# Time series analysis of dosimetric changes in target volumes and organs at risk monitored by cone beam computed tomography during radiotherapy for non-small-cell lung cancer

**DOI:** 10.1016/j.phro.2025.100822

**Published:** 2025-08-11

**Authors:** Chitchaya Suwanraksa, Wathanya Sukcharoen, Saranya Phuakphiuwong, Sittinee Kooptisirirat, Kantida Krutsuwan, Chanakran Nantasri, Apisit Jakkrit, Thanarpan Peerawong, Ponlagrit Kumwichar

**Affiliations:** a Department of Radiology, Faculty of Medicine, Prince of Songkla University, Songkhla, Thailand; b Department of Epidemiology, Faculty of Medicine, Prince of Songkla University, Songkhla, Thailand

**Keywords:** Non-small-cell lung cancer, Cone beam computed tomography, Radiotherapy, Time-series analysis, Adaptive radiotherapy

## Abstract

**Background and Purpose:**

Technological advancements in radiotherapy have enhanced the ability to increase tumor doses while sparing normal tissues. We performed a time-series cluster analysis to characterize dynamic variations in dosimetry during radiotherapy.

**Materials and Methods:**

We analyzed dosimetric variations of 40 patients with non-small-cell lung cancer who received 60 Gy over 30 daily cone beam computed tomography-guided fractions. Percentage deviations from planned doses across all fractions were calculated for organs at risk (OARs) (lungs, heart, spinal cord, and esophagus), gross tumor volume, clinical target volume (CTV), and planning target volume. K-means clustering with dynamic time-warping distances was applied to identify temporal dose patterns. The target volume with the lowest variance (95 % dose coverage of the CTV [CTV_D95_]) was selected as a reference. OAR dose deviations were clustered using the elbow method. Mean temporal trends were plotted with 95 % confidence intervals.

**Results:**

The CTV_D95_ remained stable across all fractions and all patients and was used as a reference for time-series clustering. Cluster 1 included the majority of patients and exhibited low variability and predictable dose trajectories with gradual increases in the lung and esophagus doses and stable patterns in the heart and spinal cord. Cluster 2 showed high inter-fractional variability with progressively increasing OAR dose deviations.

**Conclusions:**

Time-series clustering enabled early identification of patients with distinct dose evolution patterns. The stable trajectories of Cluster 1 may serve as internal references for adaptive radiotherapy. Variability in Cluster 2 underscores the need for routine dose monitoring to support timely offline adaptation.

## Highlights

• Time-series clustering identified dosimetric variations in lung cancer treatment.
• Early detection of dose deviations can trigger adaptive radiotherapy interventions.
• Dose deviation thresholds are essential to optimize adaptive radiotherapy protocols.

## Graphical abstract

**Figure:**
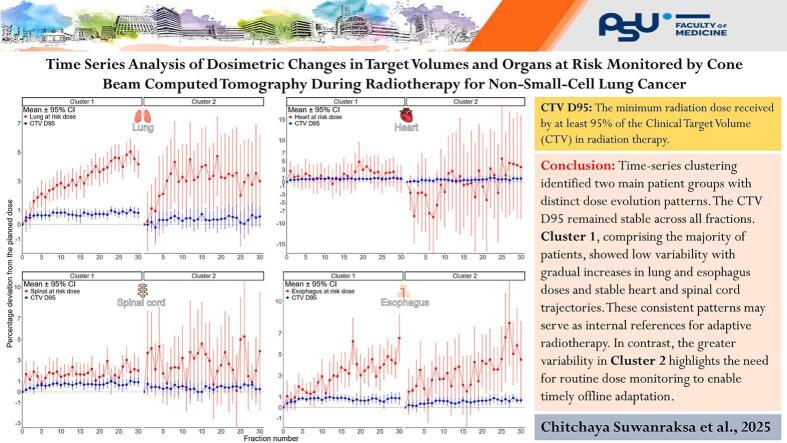

## 1 Introduction

Radiotherapy (RT) has become important for treating non-small-cell lung cancer (NSCLC) [1] by maximizing tumor dose while minimizing exposure of nearby organs at risk (OARs) [2]. Recent advances in RT, like volumetric-modulated arc therapy and adaptive RT (ART), have improved tumor dosing while sparing normal tissue [3,4].

Traditional RT assumes stable anatomy, but significant anatomical changes during treatment can affect accuracy and outcomes [3,5]. Cone-beam computed tomography (CBCT) integrated with linear accelerators enables daily image guidance by comparing patient positioning to planning CT (pCT) for accurate delivery [6]. Deformable image registration (DIR) converts CBCT to virtual CT (vCT), addressing HU limitations and enabling dose calculation in ART workflows [7,8]. ART addresses both dynamic anatomical and static changes during RT, causing variable dosimetric effects and requiring plan adjustments [5,9]. This patient heterogeneity complicates optimal ART timing [10,11].

Despite advances in RT, the link between OAR dose variations and target volume dosing remains poorly defined due to its complexity. Clarifying this relationship is key to optimizing ART, reducing OAR toxicity, and ensuring adequate target coverage [[12], [13], [14], [15]].

Offline ART workflows, which rely on periodic cumulative assessments rather than daily plan adaptation, are particularly vulnerable to persistent undetected dose deviations. To address this gap, we aimed to establish a framework for detecting and characterizing daily dose deviations throughout the course of RT.

Specifically, we compared both volumetric and dosimetric parameters derived from daily CBCT-based vCT datasets with those derived from the pCT using DIR to enhance anatomical alignment and improve dose calculation accuracy. Key structures such as target volumes (gross tumor volume [GTV], clinical target volume [CTV], and planning target volume [PTV]) and OARs (lungs, heart, esophagus, and spinal cord), as well as corresponding dosimetric parameters, including the 95 % dose coverage (D_95_) for targets and mean dose (D_mean_) and maximum dose (D_max_) for OARs, were analyzed.

Linear regression was applied independently to volumetric and dosimetric parameters to assess temporal trends throughout the treatment course. Additionally, we investigated whether specific dosimetric parameters, particularly D95, for target volumes could serve as surrogates for capturing dose variations in adjacent OARs. To characterize interfractional dynamics, time-series clustering was applied using dosimetric data alone to identify common trajectories of dose deviations. This framework may support individualized offline ART decisions by indicating when plan adaptation is most likely to improve treatment accuracy and clinical outcomes.

## 2 Materials and methods

### 2.1 Participants

This retrospective study included 40 consecutively treated patients with NSCLC who underwent RT at Songklanagarind Hospital, Thailand, using the Varian TrueBeam STx LINAC platform (Varian Medical Systems, Inc., Palo Alto, CA, USA) between December 2018 and July 2023. We included patients who underwent computed tomography (CT) (SOMATOM go.Open Pro; Siemens Healthineers AG, Eschborn, Germany) with daily CBCT fractions during RT at a prescribed dose of 60 Gy in 30 fractions. This study was approved by the Office of Human Ethics Committee, Faculty of Medicine, Prince of Songkla University (approval number: REC 66-336-7-2).

### 2.2 Patient characteristics

The characteristics of the 40 patients with NSCLC are presented in Table 1. Most patients had locally advanced nonmetastatic disease, and all underwent concurrent chemoradiotherapy. The target volume sizes varied widely across patients.

**Table 1:** Clinical characteristics of the patients.

Characteristics	Value
Patients, N	40
Sex, n (%)	
Male	31 (77.5 %)
Female	9 (22.5 %)
Age, mean ± SD	62 ± 21
BMI, mean ± SD	22.8 ± 4.4
TNM classification, n (%)	
Primary tumors	
T1	2 (5 %)
T2	8 (20 %)
T3	9 (22.5 %)
T4	21 (52.5 %)
Regional lymph nodes	
N0	5 (12.5 %)
N1	7 (17.5 %)
N2	18 (45 %)
N3	10 (25 %)
Metastasis	
M0	40 (100 %)
M1	0
Treatment, n (%)	
Concurrent chemoradiotherapy	40 (100 %)
Other	0
Targets volume (cm^3^), mean ± SD	
GTV	287.5 ± 208.5
CTV	496.8 ± 282.5
PTV	730.5 ± 354.3

BMI, body mass index; CTV, clinical target volume; GTV, gross tumor volume; PTV, planning target volume; SD, standard deviation.

### 2.3 Setting

RT was administered from Monday to Friday. To allow normal tissue repair and minimize side effects while preserving treatment effectiveness, RT was not administered during weekends (Saturdays and Sundays).

### 2.4 Image acquisition and radiation treatment planning

All patients underwent four-dimensional CT with a 3 mm slice thickness. The data were used to reconstruct the average intensity projection and maximum intensity projection images. The average intensity projection images were used to delineate the OARs, whereas maximum intensity projection images were specifically used to delineate the internal GTV, which included both the primary tumor and lymph nodes, to account for and evaluate respiratory motion during the imaging process. A 5 mm isotropic margin was added to the internal GTV to define the CTV, which was cropped to exclude bones and large vessels. For both the primary tumor and lymph nodes, the CTV was expanded by 5 mm to form the PTV. Treatment planning included volumetric-modulated arc therapy and a 6 MV photon beam performed using Eclipse treatment planning version 16.1 (Varian Medical Systems, Inc., Palo Alto, CA, USA).

### 2.5 Virtual CT generation and contour propagation

Images obtained during pCT were aligned with all CBCT images using the DIR algorithm of Velocity 4.1.4 (Varian Medical System, Inc., Palo Alto, CA, USA). Registration began with rigid alignment using matched coordinates derived from the patient’s actual treatment position recorded during CBCT acquisition, thereby ensuring alignment with the treated anatomy, followed by deformable registration using the Elastic B-Spline model. The HU values obtained during pCT were mapped onto the CBCT anatomy image to generate a new volume, which was referred to as the vCT image. Contours obtained during pCT were propagated and adjusted to fit the CBCT anatomy image for each fraction (GTV, CTV, PTV) and covered OARs such as the esophagus, lungs, heart, and spinal cord. All propagated contours were verified by clinical experts. Manual corrections of propagated contours were performed when automatic adjustments were inadequate to ensure anatomical accuracy and clinical consistency across fractions.

### 2.6 Quantification of interfractional volumetric and dose-volume changes

After generating vCT images using the DIR method, the original treatment plan was recalculated for each vCT image while preserving the monitor units and beam parameters. This enabled estimation of the actual dose delivered for each treatment fraction across the entire 30-fraction course for all 40 patients. All data were extracted from the Eclipse treatment planning system. Volumetric changes of target volumes (GTV, CTV, and PTV) and OARs (lungs, esophagus, heart, and spinal cord) were evaluated. Dosimetric parameters included the D_95_ for target volumes and the mean or maximum dose for OARs. These volumetric and dosimetric values were recorded for every treatment fraction for each patient. Deviations were assessed by comparing the value of each fraction to the planned value and expressed as the percentage change from baseline.

### 2.7 Regression and time-series analysis

Descriptive statistics were used to summarize demographics. For each treatment fraction, deviations in volumetric and dosimetric parameters from the corresponding pCT values were expressed as percentage changes. We initially analyzed the temporal patterns of dosimetric variations using linear regression, irrespective of its underlying assumptions. To reduce the influence of high-leverage observations on model estimation, outliers did not exceed 74 of 1200 data points (6.2 %) for each parameter identified at the fraction level using Cook’s distance [16] and were excluded from the regression analysis to enhance model robustness. The overall dose deviation trends remained consistent before and after exclusion. To further investigate variations across all individuals, we used generalized linear mixed models with the fraction number as a fixed effect and individuals as random effects. This approach allowed us to quantify the deviation rate over time from fraction 1 to fraction 30 and estimate the corresponding P-values. Multiple comparisons were accounted for by applying the Bonferroni correction [17] and adjusting the threshold for significance according to the number of tests performed. The earliest fraction with a significant deviation marked a shift in the overall trend.

A time-series cluster analysis was used to group time-series data of the dosimetric change (a dose of 95 % of the volume) compared to the target volumes (including the GTV, CTV, and PTV) based on their patterns over the course of the treatment period. This allowed for exploration of individual-level temporal characteristics and identification of typical deviation trajectories. To quantify the uniqueness of each patient's time-series pattern, we calculated a metric termed “distinctiveness,” which represents the average dissimilarity of one time series relative to all others in the cohort. These distinctiveness values were computed using dynamic time-warping distances; this computation method aligned time series by nonlinearly stretching or compressing the time axis to find the best match between sequences [[18], [19], [20]]. Subsequently, k-means clustering was applied to the matrix of dynamic time-warping distances to quantify the distinctiveness of each time series. Among the dosimetric measures of target volumes, the one with the lowest variance in distinctiveness across all 40 patients was selected as the representative dosimetric measure of the target volume and reflected a more stable and generalizable temporal feature. Next, k-means clustering was performed for two-dimensional time series composed of the selected dosimetric measures of the target volume and each OAR dose variation. This step aimed to reduce heterogeneity in temporal profiles and identify common patterns across patients. The optimal number of clusters (k) was identified using the elbow method on a scree plot that displayed the total within-cluster sum of squares as a function of the number of clusters. To compensate for the relatively small sample size, the optimal number was conservatively increased by one (k + 1). Clusters with fewer than 10 individuals were considered outliers and excluded from further analyses. Finally, for each remaining cluster, mean temporal trajectories were plotted across treatment fractions along with 95 % confidence intervals (CIs) and computed using the standard normal approximation [21].

Analyses were performed using the tidyverse (version 2.0.0) [22], lme4 (version 1.1.34) [23], dtwclust (version 6.0.0) [24], and cluster (version 2.1.4) [25] packages of R Language and Environment version 4.1.1 (R Foundation for Statistical Computing, Vienna, Austria).

## 3 Results

### 3.1 Linear trends of volumetric and dose-volume data

Table 2 shows the average percentage change per fraction and the earliest significant deviation from planning values.

**Table 2:** Volumetric and dose-volume parameters.

Volumetric parameter	Volumetric deviation compared to pCT parameters			Dosimetric parameters	Dosimetric deviation compared to pCT parameters		
	Average volumetric deviation %	Rate of deviation (%), mean (95 % CI)	Earliest number of fractions with significant P-values*		Average dosimetric deviation %	Rate of deviation (%), mean (95 % CI)	Earliest number of fractions with significant P-values*
GTV	−9.79	−0.33 (−0.36, −0.29)	5	GTV_D95_	0.37	0.01 (0.01, 0.02)	2
CTV	−7.14	−0.24 (−0.26, −0.21)	5	CTV_D95_	0.29	0.01 (0.01, 0.01)	2
PTV	−5.34	−0.18 (−0.20, −0.16)	7	PTV_D95_	0.65	0.02 (0.01, 0.03)	−
Lung	0.53	0.02 (0.00, −0.03)	−	Lung_Dmean_	3.87	0.13 (0.12, 0.14)	3
Esophagus	2.56	0.09 (0.07, −0.10)	−	Esophagus_Dmean_	3.40	0.11 (0.09, 0.14)	7
Heart	−1.67	−0.06 (−0.08, −0.03)	20	Heart_Dmean_	2.07	0.07 (0.02, 0.11)	−
Spinal cord	−0.38	−0.01 (−0.02, −0.01)	−	Spinal cord_Dmax_	0.57	0.02 (−0.00, 0.04)	−

*Some parameters did not demonstrate significant changes, as indicated by the absence of fractional values. CI, confidence interval; CTV, clinical target volume; D95, 95% dose coverage; D_max_, maximum dose; D_mean_, mean dose; GTV, gross tumor volume; pCT, planning computed tomography; PTV, planning target volume.

The analysis revealed notable trends in both anatomical changes and dose delivery over time. Significant negative volumetric deviations were observed in the GTV (−9.79 %), CTV (−7.14 %) starting at fraction 5, and PTV (−5.34 %) starting at fraction 7. By contrast, OARs showed no significant volumetric deviations. Small positive changes were observed in the lungs (0.53 %) and esophagus (2.56 %), and slight negative changes were observed in the heart (−1.67 %) and spinal cord (−0.38 %); these changes were not significant. Significant positive dosimetric deviations in the GTV_D95_ and CTV_D95_ of 0.37 % and 0.29 %, respectively, were observed. Regarding the mean doses of the lungs and esophagus, significant dosimetric deviations of 3.87 % and 3.40 %, respectively (with significance at fractions 3 and 7, respectively), were observed. The mean dose of the heart and maximum dose of the spinal cord did not exhibit significant deviations in dose delivery. However, these variations may be attributed to underlying nonlinear trends.

### 3.2 Time-series cluster

**Fig. 1:**
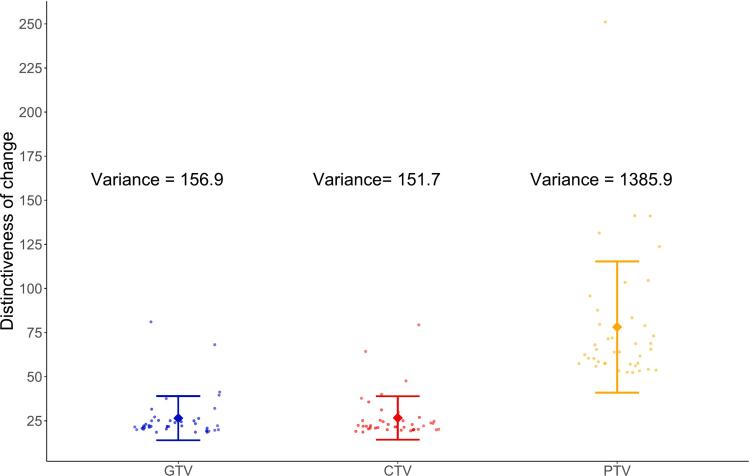
Variance of the distinctiveness of changes in the GTV_D95_, CTV_D95_, and PTV_D95._ CTV, clinical target volume; D95, 95 % dose coverage; GTV, gross tumor volume; PTV, planning target volume.

Fig. 1 illustrates the distinctiveness of changes in the GTV_D95,_ CTV_D95_, and PTV_D95_ among patients. The PTV_D95_ exhibited high variance (1385.9), thus, reflecting greater variability in response compared to those of GTV_D95_ and CTV_D95_. CTV_D95_, which demonstrated the lowest variance, was selected to represent the dosimetric changes. Stable patterns with low variance may indicate more consistent treatment effects.

**Fig. 2:**
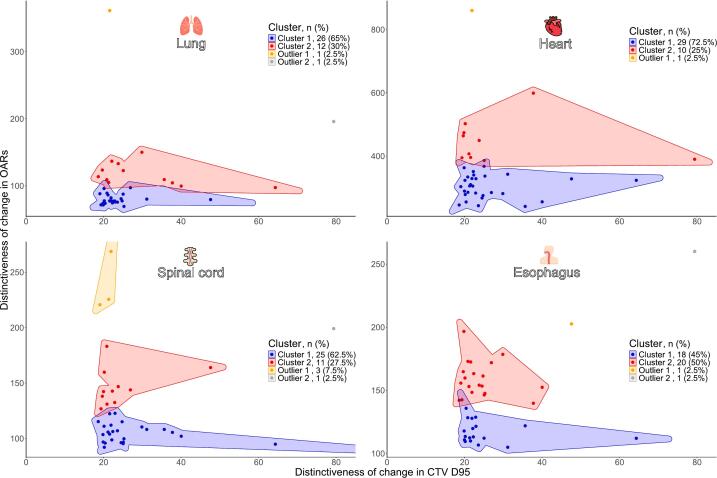
Clustering of the distinctiveness of changes in OARs relative to the CTV_D95_. CTVD_95_, 95% dose coverage of the clinical target volume; OAR, organ at risk.

Fig. 2 shows time-series clusters of changes in OARs and the CTV_D95_ over the treatment period. The analysis identified two clusters with distinct patterns of dose variability for the CTV_D95_ and OARs. Cluster 1 showed lower variability for 62.5 % to 72.5 % of patients across most OARs except the esophagus (45 %). Cluster 2 exhibited greater variability. Outliers were cases that deviated significantly from the main clusters.

**Fig. 3:**
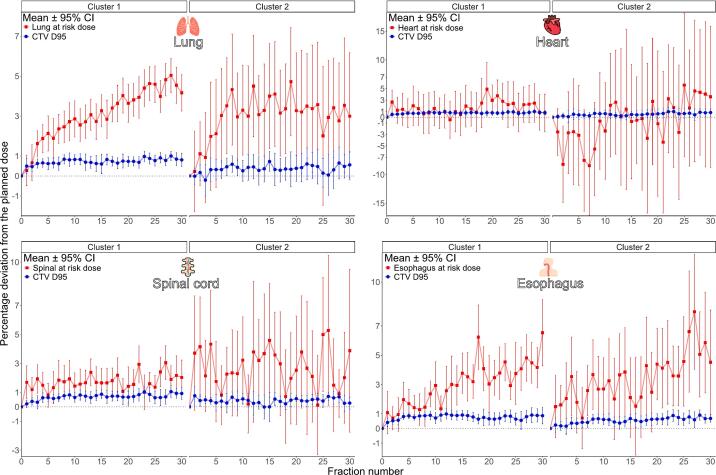
The mean percentage deviations (±95 % CIs) from the planned dose across 30 treatment fractions for each OAR, including the lung_Dmean_, heart_Dmean_, spinal cord_Dmax_, and esophagus_Dmean_, along with the CTV_D95_. Red markers denote OAR dose deviations. Blue markers represent CTV_D95_ deviations. CI, confidence interval; CTV, clinical target volume; D_95_, 95 % dose coverage; D_mean_, mean dose; OAR, organ at risk. (For interpretation of the references to colour in this figure legend, the reader is referred to the web version of this article.)

The number of clusters representing the distinctiveness of changes in OARs relative to the CTV_D95_ followed the k + 1 rule, where k was identified from the scree plots (Supplementary Figs. 1 to 4). The dynamic nature of dose evolution across clusters is presented in Fig. 3. Patients were stratified into two clusters based on time-series dose deviation trajectories. Cluster 1, which comprised the majority of patients, exhibited stable patterns with low interfractional variability and narrow 95 % CIs, thus enabling dose prediction. By contrast, Cluster 2 showed heterogeneous and fluctuating dose trajectories with wider CIs. Despite this stratification, the CTV_D95_ was consistently within 1 % of the planned dose across all fractions in both clusters, reflecting preserved target coverage throughout treatment.

Cluster 1 showed a gradual mid-treatment increase in lung dose deviation, generally within 3–5 % with narrow CIs. Cluster 2 showed earlier deviations starting around fraction 6, exceeding 5 % in the final third, indicating greater variability. Heart dose in Cluster 1 remained near zero with minimal fluctuation, while Cluster 2 showed initial underdosing and increased variability after fraction 15, exceeding ± 3 % with wider CIs. The spinal cord was stable in Cluster 1 (within ± 3 %), but Cluster 2 showed fluctuations over 5 % in later fractions with broader CIs. Esophageal deviations in Cluster 1 stayed below 5 %, with a slight rise after fraction 15. Cluster 2 showed early deviations > 3 % from fraction 4, exceeding 5 % near the end, with persistent variability.

## 4 Discussion

We identified global trends and the earliest significant deviations from planned values. Then, we identified two main clusters of time-series patterns; however, other uncommon or inconclusive patterns, possibly because of the small sample size, may have existed. Cluster 1 showed a clearly outstanding pattern with low variability. During the first 2 weeks of treatment, we could identify individuals with a Cluster 1 pattern in the lungs and esophagus who were at risk. This pattern may be used to predict the upward trend of the OAR doses and plan ART. However, Cluster 2 had high variability and other uncommon patterns (outliers); therefore, the findings were inconclusive. Cluster 1 trajectories, which were detectable with routine CBCT-based contour propagation, provided an early offline ART trigger that was especially valuable for centers without real-time adaptation.

Despite the feasibility of this study, CBCT images were acquired using standard clinical protocols without respiratory gating, which may have introduced anatomical variability, particularly in thoracic structures [26,27]. Because CBCT has low soft tissue contrast and susceptibility to respiratory and cardiac motions, its anatomical accuracy can be compromised, and the performance of DIR-based dose accumulation can be impaired [[28], [29], [30]]. The observed heart volume variability (Table 2), for example, likely reflected cardiac phase differences caused by ungated acquisition [31]. Similarly, the increased dose deviations observed in the lungs and esophagus may be more reflective of interfractional anatomical changes rather than respiratory-induced motion. This perspective aligns with previous findings of RT for NSCLC in which longitudinal anatomical alterations had a greater dosimetric impact on OARs than respiratory motion alone [32]. To mitigate these limitations, all propagated contours were reviewed by clinical experts and manually corrected to ensure interfractional consistency. Uncertainties were addressed through visual inspection, correction protocols, and interpretation of dose metrics within 95 % CIs, thus, supporting the reliability of the accumulated dose data. These mitigation strategies underscore the feasibility of CBCT-based DIR accumulation for longitudinal dose monitoring, even in the presence of intrinsic imaging limitations [33,34].

Linear regression identified clear temporal trends in both anatomical and dosimetric parameters. The GTV, CTV, and PTV showed progressive reductions over the course of treatment. Significant changes were observed as early as fractions 5 to 7, consistent with previous findings [35,36], indicating an early tumor response. Despite these anatomical regressions, target dose coverage remained stable, and deviations were maintained within 1 % of planned values, thus supporting the adequacy of current planning margins [[37], [38], [39]]. By contrast, significant increases in the lung_Dmean_ and esophagus_Dmean_ were observed beginning from fractions 3 and 7, respectively. These findings were also echoed in time-series clustering. The heart and spinal cord doses remained relatively stable, consistent with the findings of prior reports, and may reflect the anatomical stability of these structures and their relative insensitivity to respiratory-induced motion [[40], [41], [42]]. While regression captured group trends, it may miss nonlinear changes. Time-series clustering was applied to better capture interpatient variability and dose evolution.

From the time-series analysis, the stable trajectories in Cluster 1 could serve as a normative benchmark for the expected dose delivery in similar patient populations [[43], [44], [45]]. At the institutional level, such patterns could inform the development of internal thresholds to support ART decision-making. The selection of such thresholds depends on the clinical context, treatment protocols, and institutional tolerance for interfractional variability, and it requires validation prior to implementation in routine practice [13,[46], [47], [48]]. Early detection of deviations, through comparisons with the Cluster 1 reference, may enable timely interventions, such as ART planning, increased image reviews, or clinician-led reassessments.

To the best of our knowledge, no previous studies have utilized time-series cluster analyses to evaluate RT data without relying on arbitrary classifications such as expert-defined risk groupings [3,37,49]. Time-series clustering has been applied to analyze longitudinal patient data, such as biomarker responses in metastatic breast cancer [50]. Although advanced machine-learning methods provide powerful modeling capabilities, their clinical integration may be constrained by limited interpretability and resource demands [51,52]. Other statistical techniques, such as hidden Markov models, have yielded valuable insights, but they often require large datasets [53,54]. The clustering framework provides a practical stratification tool for ART planning with the current infrastructure and warrants further study in resource-constrained settings.

This study had several limitations. Clinical outcomes such as toxicity and tumor control were not assessed. Cluster 2 and outlier patterns were inconclusive because of high interpatient variability. The relatively small cohort size and limited patient diversity may have constrained the generalizability of our findings. However, the use of complete per-fraction data across 40 patients over 1200 time-resolved datapoints enabled high-resolution characterization of longitudinal dose trends, thus comparing favorably with prior interfractional studies [41,55,56]. Future studies should include clinical outcome data to examine the impact of dose deviation patterns on the treatment response and toxicity. Because of the high prevalence of anatomical changes in other tumor sites, particularly the head and neck, where offline ART is often required, applying this framework to such regions may enhance its clinical utility. These sites experience significant interfractional variability; therefore, adapting this methodology could help guide personalized ART strategies.

In conclusion, this study demonstrated the feasibility of using CBCT imaging and DIR-based dose accumulation to monitor temporal dose deviations during RT for NSCLC. Linear regression and time-series clustering offered complementary insights. Linear regression summarized group-level trends, whereas time-series clustering identified evolving interpatient dose patterns. The early-onset constant increase observed in Cluster 1 suggested the potential for internal ART benchmarks, whereas the variability in Cluster 2 highlighted the need for continued surveillance. With further validation, time-series clustering may support personalized ART strategies through data-driven adaptation in real time.

## Funding source

This research was supported by a research grant from the Faculty of Medicine, Prince of Songkla University.

## Declaration of competing interest

The authors declare that they have no known competing financial interests or personal relationships that could have appeared to influence the work reported in this paper.
