# Supplementary material for: Time series analysis of dosimetric changes in target volumes and organs at risk monitored by cone beam computed tomography during radiotherapy for non-small-cell lung cancer
Source: Phys Imaging Radiat Oncol. 2025 Aug 11;35:100822. doi: 10.1016/j.phro.2025.100822 (PMC12363579; doi:10.1016/j.phro.2025.100822)

Supplementary figures

sFigure 1. Scree plot illustrating the distinctiveness of dosimetric changes in lung-at-risk relative to CTV_{D95}.

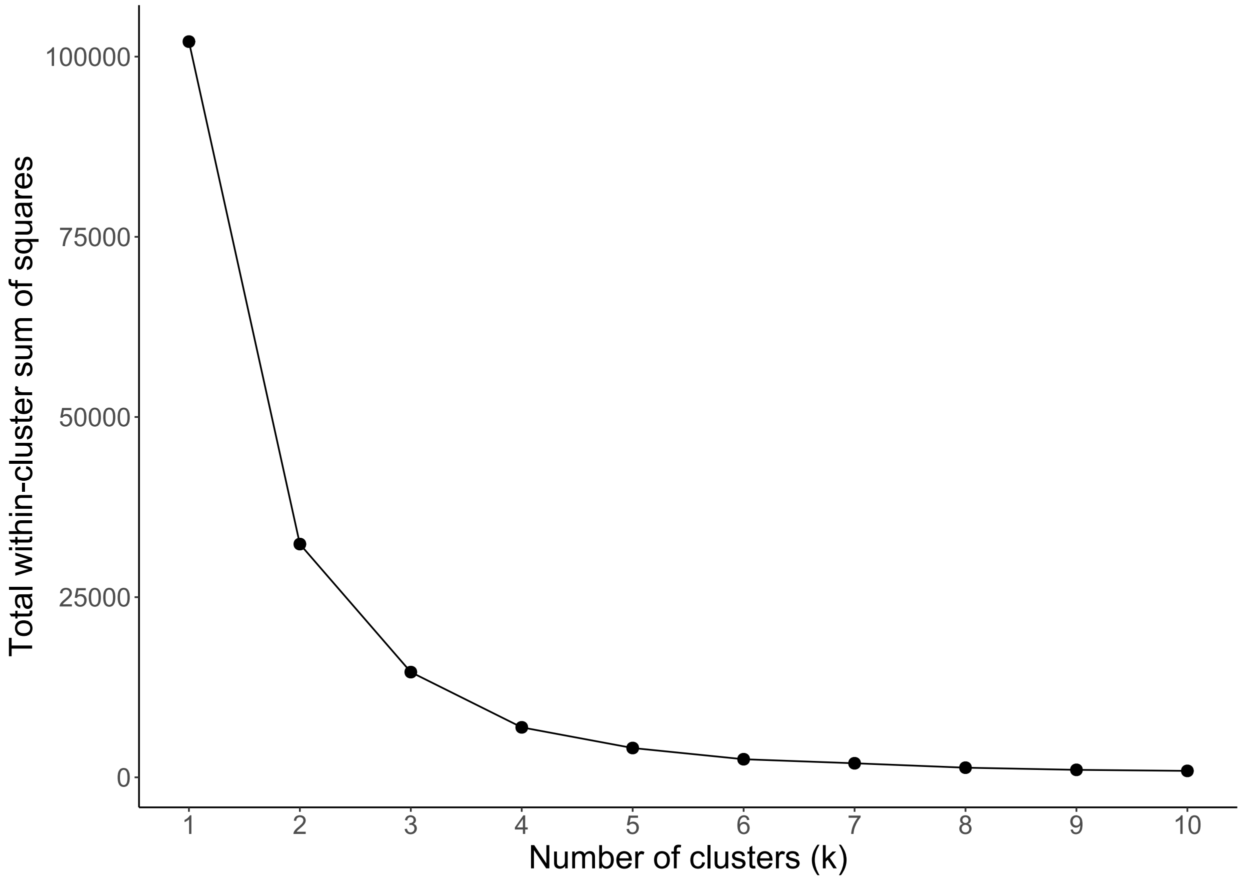

sFigure 2. Scree plot illustrating the distinctiveness of dosimetric changes in heart-at-risk relative to CTV_{D95}.

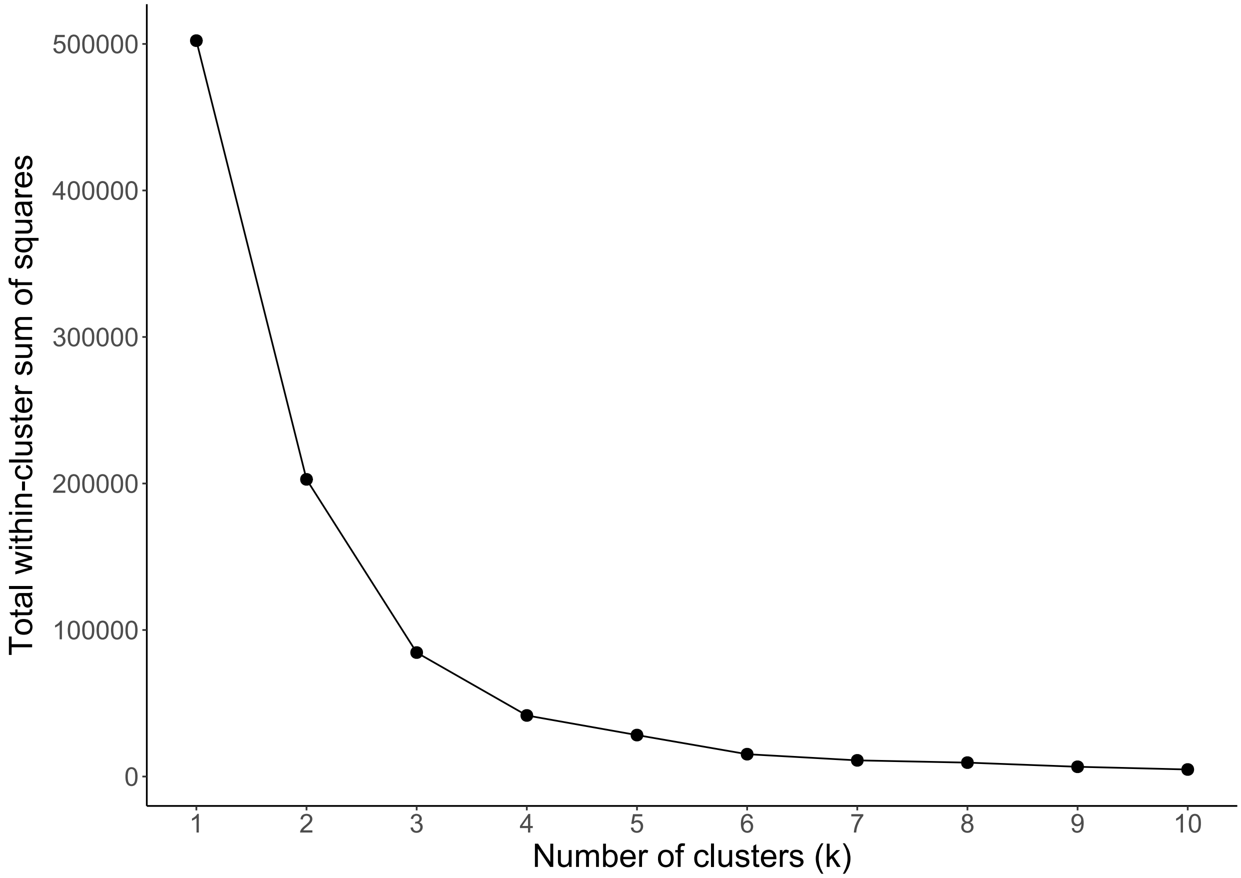

sFigure 3. Scree plot illustrating the distinctiveness of dosimetric changes in spinal cord-at-risk relative to CTV_{D95}.

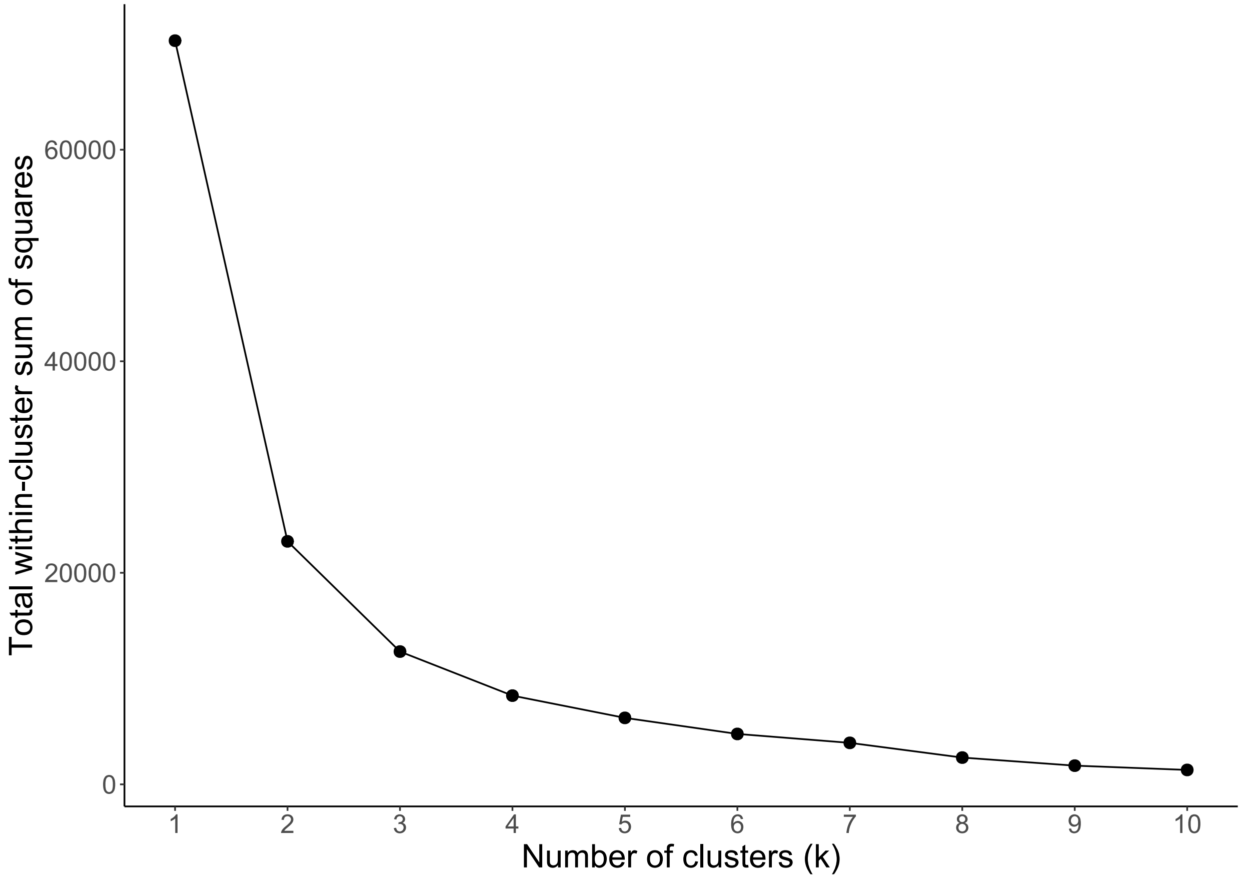

sFigure 4. Scree plot illustrating the distinctiveness of dosimetric changes in esophagus-at-risk relative to CTV_{D95}.

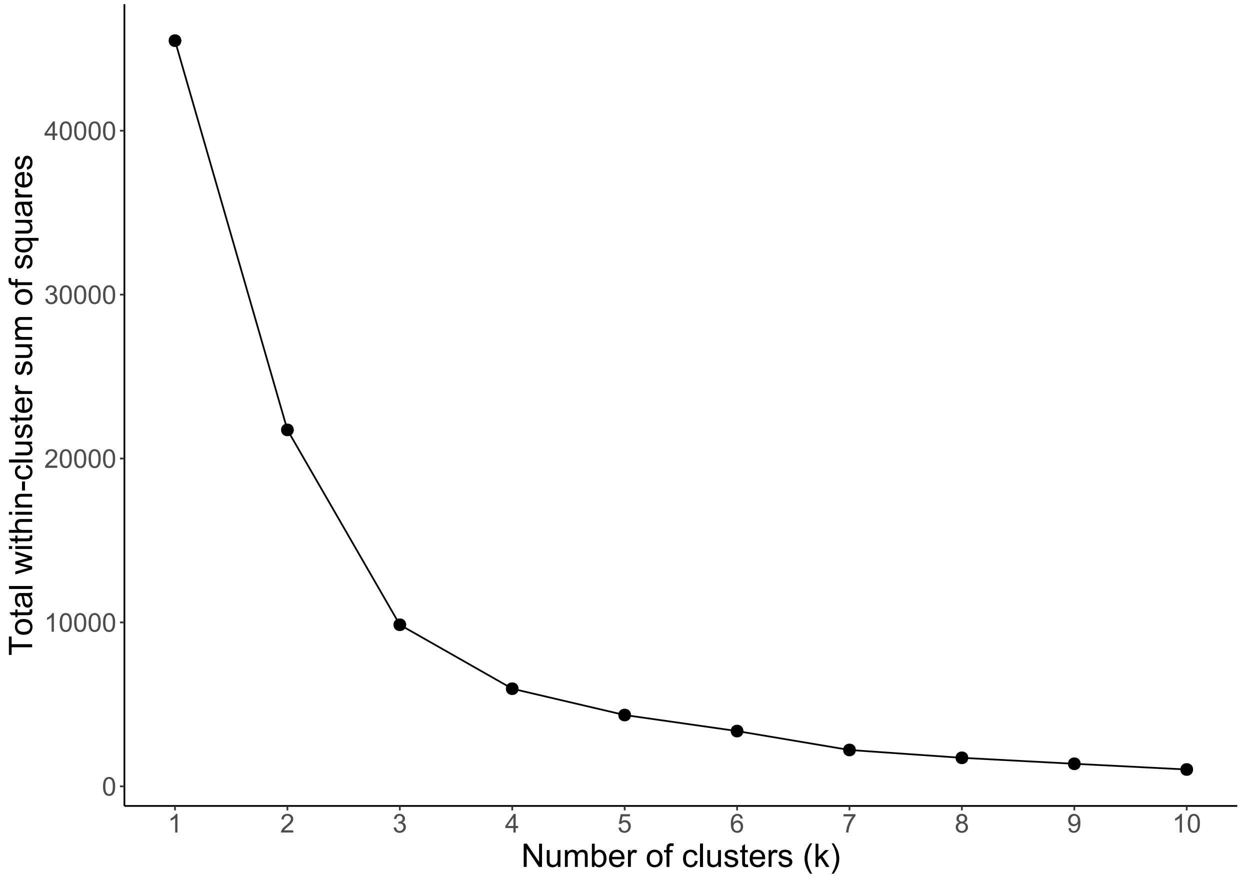

Supplement: Supplementary Data 1 [file mmc1.pdf]
